# Biomarkers of Endothelial Activation Are Associated with Poor Outcome in Critical Illness

**DOI:** 10.1371/journal.pone.0141251

**Published:** 2015-10-22

**Authors:** Carmen Mikacenic, William O. Hahn, Brenda L. Price, Susanna Harju-Baker, Ronit Katz, Kevin C. Kain, Jonathan Himmelfarb, W. Conrad Liles, Mark M. Wurfel

**Affiliations:** 1 Department of Medicine, Division of Pulmonary and Critical Care Medicine, University of Washington, Seattle, Washington, United States of America; 2 Department of Medicine, Division of Infectious Diseases, University of Washington, Seattle, Washington, United States of America; 3 Department of Biostatistics, University of Washington, Seattle, Washington, United States of America; 4 Kidney Research Institute, University of Washington, Seattle, Washington, United States of America; 5 Department of Medicine, Sandra Rotman Centre for Global Health, University Health Network-Toronto General Hospital and the Tropical Disease Unit, University of Toronto, Toronto, Ontario, Canada; King Abdullah International Medical Research Center, SAUDI ARABIA

## Abstract

**Background:**

Endothelial activation plays a role in organ dysfunction in the systemic inflammatory response syndrome (SIRS). Angiopoietin-1 (Ang-1) promotes vascular quiescence while angiopoietin-2 (Ang-2) mediates microvascular leak. Circulating levels of Ang-1 and Ang-2 in patients with SIRS could provide insight on risks for organ dysfunction and death distinct from inflammatory proteins. In this study, we determined if biomarkers of endothelial activation and inflammation exhibit independent associations with poor outcomes in SIRS.

**Methods:**

We studied 943 critically ill patients with SIRS admitted to an Intensive Care Unit (ICU) of an academic medical center. We measured plasma levels of endothelial markers (Ang-1, Ang-2, soluble vascular cell adhesion molecule-1 (sVCAM-1)) and inflammatory markers (interleukin-6 (IL-6), interleukin-8 (IL-8), granulocyte-colony stimulating factor (G-CSF), soluble tumor necrosis factor receptor-1 (sTNFR-1)) within 24 hours of enrollment. We tested for associations between each marker and 28 day mortality, shock, and day 3 sequential organ failure assessment (SOFA) score. For 28 day mortality, we performed sensitivity analysis for those subjects with sepsis and those with sterile inflammation. We used multivariate models to adjust for clinical covariates and determine if associations identified with endothelial activation markers were independent of those observed with inflammatory markers.

**Results:**

Higher levels of all biomarkers were associated with increased 28 day mortality except levels of Ang-1 which were associated with lower mortality. After adjustment for comorbidities and sTNFR-1 concentration, a doubling of Ang-1 concentration was associated with lower 28 day mortality (Odds ratio (OR) = 0.81; p<0.01), shock (OR = 0.82; p<0.001), and SOFA score (β = -0.50; p<0.001), while Ang-2 concentration was associated with increased mortality (OR = 1.55; p<0.001), shock (OR = 1.51; p<0.001), and SOFA score (β = +0.63; p<0.001). sVCAM-1 was not independently associated with SIRS outcomes.

**Conclusions:**

In critically ill patients with SIRS, early measurements of Ang-1 and Ang-2 are associated with death and organ dysfunction independently of simultaneously-measured markers of inflammation.

## Introduction

The systemic inflammatory response syndrome (SIRS) is an early manifestation of a variety of forms of critical illness [1]. Sepsis, defined by clinical suspicion for infection in combination with SIRS, is the predominant cause for intensive care unit (ICU) admission in the developed world and is the leading cause of death in non-cardiac ICUs [2]. SIRS can also develop in the absence of infection in response to other causes such as trauma and pancreatitis.

Classically, SIRS has been thought to be a manifestation of early innate immune activation followed by a dysregulated inflammatory response [3]. Several pro-inflammatory cytokines and acute phase reactants have been associated with poor outcomes in sepsis [4–12]. In particular, plasma levels of IL-6, IL-8, and G-CSF have been previously shown to be associated with mortality in sepsis in humans [13–15]. sTNFR-1 has been shown to be more highly associated with mortality than other pro-inflammatory biomarkers [16]. These biomarkers are all thought to participate in various components of the early patient responses to sepsis: early innate immune responses (IL-6, sTNF-R1) and inflammatory cell differentiation and recruitment (G-CSF and IL-8). While the data supporting the role of pro-inflammatory cytokines in sepsis is strong, multiple trials using broad and targeted anti-inflammatory therapies have not been successful in improving clinical outcomes, suggesting a need to rethink this model of SIRS pathophysiology [17].

More recently, interest has focused on the role of the endothelium in mediating the host response in SIRS [18–21]. Angiopoietin-1 (Ang-1) and angiopoietin-2 (Ang-2) are secreted endothelial growth factors with opposing roles in stabilizing endothelium or promoting vascular leak respectively [22]. Expressed primarily by pericytes and platelets, Ang-1 stabilizes the endothelium, inhibits vascular leakage and suppresses inflammatory and coagulation gene expression through constitutive activation via phosphorylation of its cognate receptor, Tie-2. In contrast, Ang-2 is expressed and rapidly released by endothelial cells upon activation. Ang-2 functions to promote loss of barrier integrity by sensitizing endothelium to stimuli that disrupt microvascular integrity, resulting in vascular leak, a major mechanism of organ damage [22]. In patients with sepsis, we and others have shown that high Ang-2 and low Ang-1 are associated with poor clinical outcomes [23–30]. With activation of the endothelium, luminal adhesion molecules are upregulated and enter the circulation in soluble form, including soluble vascular cell adhesion molecule-1 (sVCAM-1). Circulating levels of adhesion molecules have been linked to poor outcomes in SIRS and sepsis [31,32].

While there have been several small studies linking these endothelial activation markers with sepsis outcomes it remains to be shown that these associations can be observed in a larger and broader set of critically ill patients with SIRS. Furthermore, there have been no studies that have simultaneously measured markers of endothelial activation and markers of inflammation to determine the extent to which associations between these two marker classes and outcome are independent of each other. Here we report studies in a large cohort (n = 943) of critically ill patients with SIRS in which we have simultaneously measured circulating levels of pro-inflammatory cytokines and chemokines (IL-6, IL-8, G-CSF, sTNFR-1) and markers of endothelial activation/dysregulation (Ang-1, Ang-2, sVCAM-1) in plasma obtained within 24 hours of presentation. We hypothesized that biomarkers of endothelial activation and dysfunction are associated with poor outcomes in patients with SIRS independently from circulating markers of inflammation.

## Materials and Methods

### Subjects

The Harborview Medical Center (Seattle, WA) SIRS cohort includes subjects meeting at least 2 of 4 SIRS criteria [33]. This cohort has been described previously [34]. Briefly, subjects were consecutively enrolled upon admission to the ICU with SIRS after exclusion of patients with major trauma, intracranial hemorrhage, HIV, immunosuppression, or a current diagnosis of cancer. Due to the fact this cohort was previously used for genetic studies, subjects were limited to Caucasians. Plasma specimens were obtained from subjects within 24 hours of admission to the ICU in tubes anti-coagulated with EDTA. Survivors were those alive or discharged within 28 days. This study was approved by the University of Washington human subjects institutional review board prior to the initiation of the study. These samples were obtained under a waiver of consent and subject’s clinical and personal data were anonymized and de-identified prior to analysis.

### Protein Measurement

Plasma samples were thawed no more than once and concentrations of IL-6, IL-8, G-CSF, sTNFR-1, Ang-1, Ang-2, and sVCAM-1 were measured at the same time using a multiplex immunoassay (Meso Scale Discovery, Rockville, MD). Samples were diluted to fit within the dynamic range of the assay: IL-6, IL-8, sTNFR-1, G-CSF (0.08pg/mL to 2500pg/mL); Ang-1 (3pg/mL to 100,000pg/mL); Ang-2 (0.5pg/mL to 10,000pg/mL); and sVCAM-1 (0.05pg/mL to 1000pg/mL). Samples that fell below the lower limit of detection or above the upper limit of detection were assigned those values, respectively.

### Statistical Analyses

Our primary analysis tested the association between biomarkers and inpatient mortality to day 28. To compare concentrations of biomarkers between those alive and dead at 28 days, and those with sepsis and sterile inflammation we used a Mann-Whitney U test. Given the skewed distribution of the biomarker concentrations, models were evaluated with the exposure variable transformed to the log base 2 and presented per doubling. To test for associations with 28-day mortality, we first used unadjusted logistic regression. We then created a multi-variable logistic model to control for demographic or clinical characteristics that may have affected these outcomes including age, gender, location of admission (medical versus surgical ICU), source of admission (outside hospital versus emergency room), body mass index, smoking status, diabetes, chronic renal insufficiency, cirrhosis, the presence or absence of infection and severity of illness by Acute Physiology and Chronic Health Evaluation (APACHE) III score [35]. In order to determine whether endothelial biomarkers were associated with mortality independently of biomarkers of inflammation, we further added log-base 2 transformed IL-6 or sTNFR1 concentrations to the multivariate model. For patients presenting with sepsis or sterile inflammation, we performed a pre-specified sensitivity analysis using the same multivariate models to detect associations with 28 day mortality. Correlations between biomarkers were assessed using a Spearman’s rank-order correlation.

In secondary analyses, we tested for association between biomarkers and development of other poor clinical outcomes including shock and organ dysfunction. In line with general guidelines, shock was defined by the occurrence of two measurements of hypotension (systolic blood pressure < 90mmHg) separated by more than 30 minutes and less than 120 minutes *and* at least 500mL of intravenous fluid administration during this time period, or administration of any dose of vasopressor at any time [1]. We used logistic regression to identify associations in unadjusted and multivariate models. We also tested for associations between biomarkers and organ dysfunction as measured by day 3 Sequential Organ Failure Assessment (SOFA) score in those who survived to day 3 [36]. We used multiple linear regression using unadjusted models and models adjusted for clinical covariates and inflammatory biomarker concentrations. Statistical analyses were performed with R version 3.0.0 [37].

## Results

We measured plasma biomarkers in 943 subjects admitted to the ICU at Harborview Medical Center (Seattle, WA) who met SIRS criteria (Table 1). These subjects had an average age of 55 years and were predominantly male (64%). The 28 day mortality was 12% (N = 110) and on enrollment sepsis was the main cause of SIRS (66%). Subjects who died at 28 days were more likely to have cirrhosis and chronic renal insufficiency and had higher APACHE III scores. Of these 110 subjects, 39 had acute respiratory distress syndrome and 40 had acute kidney injury [38,39].

**Table 1:** Subject Characteristics.

Characteristics	Alive at 28 days (N = 833)	Dead at 28 days (N = 110)
Patient Age, mean± SD	55.1 ± 16.1	57.4 ± 18.1
Male patients, no. (%)	532 (63.9%)	69 (62.7%)
Caucasian, no. (%)	833 (100%)	110 (100%)
Source of ICU admit, no. (%)		
Medical	443 (53.2%)	73 (66.4%)
Surgical	390 (46.8%)	37 (33.6%)
Source of Critical Illness, no. (%)		
Sepsis	546 (65.5%)	83 (75.5%)
Pneumonia	169 (20.3%)	24 (21.8%)
Trauma	0 (0%)	0 (0%)
Other	152 (18.2%)	25 (22.7%)
APACHE III, mean ± SD	47.8 ± 24.1	74.1 ± 29.7
Comorbidities, no. (%)		
Diabetes	205 (24.6%)	28 (25.5%)
Cirrhosis	64 (7.7%)	20 (18.2%)
Chronic Renal Insufficiency	50 (6.0%)	15 (13.6%)
Smoking	485 (58.2%)	53 (48.2%)
BMI, mean ± SD	30.2 ± 10.2	32.2 ± 12.5

SD = Standard Deviation; APACHE III = Acute Physiology and Chronic Health Evaluation III; BMI = Body Mass Index

The production of inflammatory and endothelial biomarkers may be similarly regulated. In order to test whether biomarkers within or across categories were correlated, we performed Spearman correlations between each individual biomarker. In general, biomarkers of endothelial activation and inflammation were only modestly correlated with one another with correlation coefficients generally less than 0.5 (S1 Table).

### Associations with 28 Day Mortality

All biomarkers tested were significantly different between those alive and dead at 28 days (Table 2). Survivors had lower levels of both pro- and anti-inflammatory biomarkers compared to non-survivors (median (pg/ml)): IL-6 (116 vs 260; p<0.001), IL-8 (13 vs 31; p<0.001), G-CSF (27 vs 35; p<0.001), and sTNFR-1 (7719 vs 18197; p<0.001). For the endothelial biomarkers, Ang-1 was higher in survivors (5719 pg/ml) versus non-survivors (2504 pg/ml; p<0.001; Table 2), while Ang-2 was lower in survivors (11934 pg/ml) versus non-survivors (42063 pg/ml; p<0.001). sVCAM-1 was also lower in survivors (536 pg/ml) versus non-survivors (819 pg/ml; p<0.001; Table 2).

**Table 2:** Comparison of Plasma Biomarkers and 28 day mortality.

Biomarkers (pg/mL)	Subjects(N)	Alive at 28 Days, Median (IQR)	Dead at 28 Days, Median (IQR)	p ^a^
Inflammation:				
IL-6	888	116 (53, 277)	260 (104, 757)	1.8x10^-08^
IL-8	888	13 (7, 25)	31 (13, 79)	1.4x10^-11^
G-CSF	888	27 (16, 52)	35 (18, 127)	5.2x10^-03^
sTNFR-1	888	7719 (5154, 13015)	18197 (11302, 31017)	1.4x10^-19^
Endothelial Activation:				
Ang-1	930	5719 (2642, 10123)	2504 (1417, 5760)	3.7x10^-09^
Ang-2	939	11934 (6843, 24641)	42063 (17094, 76983)	2.8x10^-18^
Ang-2/Ang-1	930	2.3 (0.8, 7.3)	15.6 (4.0, 60.4)	9.1x10^-17^
sVCAM-1	939	536 (422, 756)	819 (553, 1249)	7.0x10^-14^
Comparison				
Apache III	939	44 (30, 62)	72 (52, 96)	2.5x10^-18^

IQR = Interquartile Range; IL-6 = Interleukin-6; IL-8 = Interleukin-8; G-CSF = Granulocyte colony stimulating factor; sTNFR-1 = Soluble Tumor Necrosis Factor Receptor-1; Ang-1 = Angiopoietin-1; Ang-2 = Angiopoietin-2; sVCAM-1 = Soluble Vascular Adhesion Molecule-1.

^*a*^ P value for Mann-Whitney U Test

We then used multiple logistic regression to determine the relationship between log_2_ -transformed biomarker concentrations and 28-day mortality. We found strong associations between all biomarkers and mortality in unadjusted and models adjusted for demographics/comorbidities (age, gender, admitting service, source of admission, BMI, tobacco use, diabetes, chronic renal insufficiency, cirrhosis, and presence of infection; Table 3). Because APACHE III score obtained on admission to an ICU is a robust predictor of in-hospital mortality [35], we added it to the multivariate logistic regression model to determine if associations between biomarker levels and mortality were independent of severity of illness. In the demographic/comorbidity and APACHE III adjusted model, a doubling of the concentration of each biomarker was associated with an increased risk for 28 day mortality with the exception of Ang-1 which was associated with protection from 28 day mortality (Odds Ratio (95% confidence interval); OR 0.76 (0.66–0.88); p<0.001). On an individual basis, the relative amount of Ang-2 to Ang-1 produced may be relevant [40]. We tested for association between the ratio of Ang-2/Ang-1 and mortality and found that with each doubling of the Ang-2/Ang-1 ratio, there was increased risk for mortality after adjustment for demographics/comorbidities and APACHE III (OR 1.34 (1.19–1.50); p<0.001). In this same multivariate model, the highest odds ratio amongst the biomarkers for death at 28 days was for sVCAM-1 (OR 2.56 (1.76–3.73); p<0.001).

**Table 3:** Multivariate Analysis of Biomarker Association with 28 Day Mortality.

	Unadjusted		Adjusted^a^		APACHE III Adjusted ^b^		IL-6 Adjusted ^c^		sTNFR-1 Adjusted ^d^	
Biomarkers	OR (95% CI)	p^e^	OR (95% CI)	p	OR (95% CI)	p	OR (95% CI)	p	OR (95% CI)	p
Inflammation:										
IL-6	1.37 (1.25, 1.51)	1.3x10^-10^	1.41 (1.27, 1.57)	6.6x10^-11^	1.29 (1.15, 1.45)	1.0x10^-5^				
IL-8	1.47 (1.32, 1.63)	5.3x10^-13^	1.47 (1.32, 1.64)	7.0x10^-12^	1.44 (1.26, 1.64)	6.9x10^-8^				
G-CSF	1.21 (1.10, 1.34)	1.3x10^-4^	1.24 (1.12, 1.38)	5.3x10^-5^	1.13 (1.01, 1.26)	3.0x10^-2^				
sTNFR-1	2.34 (1.95, 2.81)	3.3x10^-20^	2.28 (1.85, 2.79)	3.2x10^-15^	1.96 (1.57, 2.44)	5.4x10^-9^				
Endothelial Activation:										
Ang-1	0.69 (0.61, 0.78)	1.2x10^-9^	0.71 (0.62, 0.81)	2.5x10^-7^	0.76 (0.66, 0.88)	2.1x10^-4^	0.74 (0.64, 0.86)	9.1x10^-5^	0.81 (0.71, 0.93)	2.4x10^-3^
Ang-2	2.09 (1.75, 2.49)	4.1x10^-16^	2.06 (1.68, 2.54)	7.3x10^-12^	1.72 (1.37, 2.15)	5.6x10^-6^	1.77 (1.40, 2.23)	1.5x10^-6^	1.55 (1.22, 1.96)	3.5x10^-4^
Ang-2/Ang-1	1.48 (1.35, 1.62)	6.8x10^-17^	1.47 (1.32, 1.63)	7.3x10^-13^	1.34 (1.19, 1.50)	1.1x10^-6^	1.36 (1.21, 1.53)	1.8x10^-7^	1.26 (1.13, 1.42)	5.2x10^-5^
sVCAM-1	2.63 (2.07, 3.36)	5.0x10^-15^	2.71 (1.94, 3.78)	5.7x10^-9^	2.56 (1.76, 3.73)	1.0x10^-6^	2.46 (1.69, 3.59)	2.8x10^-6^	1.37 (0.92, 2.06)	NS

OR = Odds Ratio per doubling; CI = Confidence Interval; APACHE III = Acute Physiology and Chronic Health Evaluation III.

^*a*^ Logistic regression adjusted for age, gender, presence of infection, admitting service (medical vs. surgical), source of admission (outside hospital vs. emergency room), body mass index, smoking status, diabetes mellitus, chronic renal insufficiency, and cirrhosis

^*b*^ Adjusted for APACHE III and covariates in ^a^.

^*c*^ Adjusted for Log_2_(IL-6) concentration and covariates in ^a^.

^*d*^ Adjusted for Log_2_(sTNFR-1) concentration and covariates in ^a^.

^e^ For the number of tests in this table, a Bonferroni p<0.05 is equivalent to p<1.56x10^-3^

In order to assess whether associations between markers of endothelial activation/dysfunction and mortality were independent of circulating markers of inflammation we tested multivariate models in which IL-6 or sTNFR-1 were included and found that Ang-1, Ang-2, and the ratio of Ang-2/Ang-1 remained associated with mortality in both models (Table 3). Associations between sVCAM-1 and mortality remained after adjustment for IL-6 levels but not sTNFR-1.

Because the pathophysiology of sepsis may be different than that of all critically ill patients with SIRS, we performed a pre-specified sensitivity analysis amongst patients presenting with sepsis (N = 632) and amongst those with sterile inflammation (N = 314). There were no major demographic or comorbidity differences in the two populations (S2 Table). Circulating levels of IL-6, IL-8, sTNFR-1, Ang-2, and sVCAM-1 were higher in those with sepsis compared to sterile inflammation (S3 Table). Amongst patients with sepsis, we again found that all biomarkers were associated with mortality in unadjusted and adjusted models for comorbidities and APACHE III scores with the exception of G-CSF (S4 Table). After adjustment for IL-6 concentration, all endothelial biomarkers remained associated with mortality. In contrast, after adjustment for concentration of sTNF-R1, Ang-2 and sVCAM-1 were no longer associated with mortality (S4 Table). In subjects with sterile inflammation, all biomarkers were associated with mortality in unadjusted and adjusted models for comorbidities and APACHE III scores with the exception of Ang-1 (S5 Table). After adjustment for IL-6 or sTNF-R1 concentrations, Ang-1 and sVCAM-1 were no longer associated with mortality (S5 Table).

### Associations with Shock and Organ Dysfunction

Amongst the 943 SIRS subjects enrolled, 216 subjects (23%) met criteria for shock. In multivariable logistic models, all biomarkers tested were associated with shock. A doubling of circulating levels of Ang-2 and sVCAM-1 were associated with increased risk of shock (OR (95%CI); Ang-2: 1.63 (1.44–1.85); sVCAM-1: 1.63 (1.26–2.10)), while levels of Ang-1 were associated with reduced risk of shock (0.77 (0.70–0.86; Table 4). After adjustment for either IL-6 or sTNFR-1 levels, the association with shock persisted for both Ang-1 and Ang-2 and the ratio of Ang-2/Ang-1 (Table 4). In contrast, associations with sVCAM-1 and shock were markedly attenuated by adjustment for IL-6 levels and extinguished by adjustment for sTNFR-1 levels (Table 4).

**Table 4:** Multivariate Analysis of Biomarker Association with Shock.

	Unadjusted		Adjusted^a^		IL-6 Adjusted^b^		sTNFR-1 Adjusted^c^	
Biomarkers	OR (95%CI)	p^d^	OR (95% CI)^a^	p	OR (95% CI)	p	OR (95% CI)	p
Inflammation:								
IL-6	1.26 (1.16, 1.36)	5.8x10^-9^	1.29 (1.19, 1.40)	5.1x10^-10^				
IL-8	1.14 (1.06, 1.23)	6.8x10^-3^	1.13 (1.04, 1.23)	4.5x10^-3^				
G-CSF	1.21 (1.12, 1.31)	2.2x10^-6^	1.22 (1.13, 1.33)	9.8x10^-7^				
sTNFR-1	1.54 (1.35, 1.76)	3.0x10^-10^	1.47 (1.27, 1.70)	3.6x10^-7^				
Endothelial Activation:								
Ang-1	0.78 (0.71, 0.86)	3.8x10^-7^	0.77 (0.70, 0.86)	1.8x10^-6^	0.81 (0.73, 0.91)	3.2x10^-4^	0.82 (0.74, 0.92)	7.4x10^-4^
Ang-2	1.64 (1.46, 1.84)	2.7x10^-17^	1.63 (1.44, 1.85)	3.2x10^-14^	1.42 (1.23, 1.64)	2.3x10^-6^	1.51 (1.30, 1.76)	8.7x10^-8^
Ang-2/Ang-1	1.31 (1.23, 1.40)	2.2x10^-16^	1.31 (1.22, 1.41)	1.3x10^-13^	1.23 (1.14, 1.33)	3.1x10^-7^	1.25 (1.15, 1.36)	1.3x10^-7^
sVCAM-1	1.62 (1.33, 1.96)	1.2x10^-6^	1.63 (1.26, 2.10)	1.6x10^-4^	1.41 (1.06, 1.87)	1.9x10^-2^	1.15 (0.84, 1.57)	NS

OR = Odds Ratio per doubling; CI = Confidence Interval; APACHE III = Acute Physiology and Chronic Health Evaluation III.

^*a*^ Logistic regression adjusted for age, gender, presence of infection, admitting service (medical vs. surgical), source of admission (outside hospital vs. emergency room), body mass index, smoking status, diabetes mellitus, chronic renal insufficiency, and cirrhosis

^*b*^ Adjusted for Log_2_(IL-6) concentration and covariates in ^a^.

^*c*^ Adjusted for Log_2_(sTNFR-1) concentration and covariates in ^b^.

^*d*^ For the number of tests in this table, a Bonferroni p<0.05 is equivalent to p<2.08x10^-3^

We also tested whether circulating biomarkers were associated with cumulative organ dysfunction on day 3 of enrollment as measured by the SOFA score [36]. The median for SOFA score on day 3 was 3 (IQR 1–5). We found that all biomarkers were associated with SOFA score in unadjusted and adjusted models (P<0.001; Table 5). For the biomarkers of endothelial activation and dysfunction in multivariate models, each doubling of Ang-1 was associated (β coeff (95%CI)) with a decrease in SOFA score of 0.67 (-0.79, -0.54), while with each doubling of Ang-2 there was an increase in SOFA score of 0.97 (0.83, 1.11). In adjusted models an increasing ratio of Ang-2/Ang-1 was also associated with an increase in the SOFA score of 0.61 (0.54, 0.69; Table 5). After adjustment for inflammation with IL-6 or sTNF-R1, all biomarkers of endothelial activation and dysfunction remained independently associated with SOFA score.

**Table 5:** Multivariate Analysis of Biomarker Association with Day 3 SOFA Score.

	Unadjusted		Adjusted β^a^		IL-6 Adjusted^b^		sTNFR-1 Adjusted β^c^	
Biomarker	β (95% CI)	p ^e^	β (95% CI)	p	β (95% CI)	p	β (95% CI)	p
Inflammation:								
IL-6	0.44 (0.34, 0.53)	4.2x10^-20^	0.46 (0.36, 0.55)	6.7x10^-21^				
IL-8	0.42 (0.30, 0.53)	2.8x10^-12^	0.31 (0.19,0.43)	1.8x10^-7^				
G-CSF	0.38 (0.27, 0.49)	2.5x10^-11^	0.41 (0.30, 0.52)	3.7x10^-13^				
sTNFR-1	1.08 (0.78, 1.37)	6.0x10^-13^	0.99 (0.70, 1.28)	3.2x10^-11^				
Endothelial Activation:								
Ang-1	-0.78 (-0.90, -0.66)	7.7x10^-39^	-0.67 (-0.79, -0.54)	6.6x10^-25^	-0.55 (-0.68, -0.43)	8.1x10^-19^	-0.50 (-0.63, -0.37)	8.0x10^-15^
Ang-2	1.09 (0.96, 1.23)	3.1x10^-59^	0.97 (0.83, 1.11)	3.5x10^-42^	0.78 (0.62, 0.94)	4.9x10^-22^	0.63 (0.46, 0.79)	2.0x10^-13^
Ang-2/Ang-1	0.69 (0.62, 0.76)	9.4x10^-82^	0.61 (0.54, 0.69)	1.2x10^-55^	0.51 (0.43, 0.60)	3.0x10^-34^	0.45 (0.36, 0.54)	7.5x10^-24^
sVCAM-1	1.78 (1.52, 2.05)	1.0x10^-38^	1.51 (1.20, 1.84)	1.1x10^-20^	1.20 (0.87, 1.53)	6.3x10^-13^	0.60 (0.21, 0.99)	2.6x10^-3^

SOFA = sequential organ failure assessment, β = Beta coefficient for increase in SOFA score with each doubling of biomarker concentration; CI = confidence interval.

^*a*^ Linear regression adjusted for age, gender, presence of infection, admitting service (medical vs. surgical), source of admission (outside hospital vs. emergency room), body mass index, smoking status, diabetes mellitus, chronic renal insufficiency, and cirrhosis

^*b*^ Adjusted for Log_2_(IL-6) concentration and covariates in ^a^.

^*c*^ Adjusted for Log_2_(sTNFR-1) concentration and covariates in ^a^.

^e^ For the number of tests in this table, a Bonferroni p<0.05 is equivalent to p<1.56x10^-3^

## Discussion

SIRS affects one third of all hospitalized patients, greater than 50% of all ICU patients, and greater than 80% of all surgical ICU patients [41]. However, only a small subset of this large population progress to end-organ failure and death. Endothelial dysfunction may play an important role in determining risks for this progression. Using a large cohort of patients admitted to medical and surgical ICUs with SIRS we have identified strong associations between biomarkers of endothelial activation/dysfunction (Ang-1, Ang-2, sVCAM-1) and 28 day mortality, shock, and organ failure. While these biomarkers have previously been shown to be associated with death in patients with sepsis or associated renal or pulmonary end-organ dysfunction [25,27,29,30,42,43], our report is the first to demonstrate such an association in a large and inclusive cohort of patients with SIRS.

Mediators of acute inflammation such as IL-6, IL-8, and TNFR-1 have long been implicated in the pathophysiology of SIRS/Sepsis [4–8,13,44]. It is thought that early production of inflammatory mediators drives the development of SIRS and organ dysfunction after a major clinical event like a severe infection. In this study we found strong associations between these biomarkers and death in patients with SIRS consistent with prior reports. We then tested whether the variability in markers of inflammation might account for the associations observed with markers of endothelial activation/dysfunction. We found that even after adjustment for inflammatory biomarker levels, associations between biomarkers of endothelial activation and mortality remained very strong. This finding demonstrates for the first time that markers of endothelial activation/dysfunction might contribute to risks for SIRS-related organ dysfunction and death in ways that are distinct from traditional markers of inflammation.

The inappropriate wide-spread activation of the endothelium in sepsis contributes to coagulopathy, increased production of vascular adhesion molecules, promotion of ongoing inflammation, and diffuse capillary leak [19]. Studies in animal models have shown that angiopoietin axis participates in mediating these effects in experimental models of sepsis [45]. Ang-1 takes part in maintaining endothelial integrity and inhibiting inflammation while Ang-2 promotes endothelial leak. Furthermore, treatments targeted in animal models of sepsis to block angiopoietin-2, or enhance angiopoietin-1 have been shown to be protective [29,46–48]. We and others have shown in smaller studies that the Ang-1/Ang-2 axis is implicated in sepsis pathogenesis and associated with poor outcomes [23,25,49]. In contrast, another study of ICU patients who had not yet met SIRS criteria, showed that Ang-1 and Ang-2 were not associated with the development sepsis [50]. This study differed from ours in that all patients met SIRS criteria on entry, suggesting that elevations in Ang-1 and Ang-2 may be temporally related to the development of SIRS. Our much larger study inclusive of patients with SIRS again shows that Ang-1 and Ang-2, and a higher Ang-2/Ang-1 ratio are highly associated with SIRS-related and sepsis-related mortality, shock and organ dysfunction. Taken together, these findings support the development of pharmacologic therapies directed at endothelial stabilization for the treatment of SIRS [51].

While previous studies have primarily focused on the role of these biomarkers in sepsis, our study also assesses the role of endothelial activation in subjects with sterile inflammation for reasons other than trauma. While we found that concentrations of the majority of the biomarkers are higher in sepsis, the associations with 28 day mortality in sterile inflammation are mostly similar. We did find that decreasing Ang-1 concentration was associated with mortality from sepsis while it was not in sterile inflammation. Conversely, G-CSF concentration was not associated with mortality in sepsis but was independently associated in sterile inflammation. Taken together, these results suggest that endothelial activation plays a role in SIRS in response to sepsis or sterile inflammation.

Activated endothelium upregulates adhesion molecules and soluble forms of these adhesion molecules are shed from the endothelium and detectable in circulating plasma. VCAM-1, a member of the immunoglobulin family allows for firm adhesion of leukocytes to the endothelium and subsequent diapedesis. Activated endothelium releases sVCAM-1, and this protein has been previously shown to be associated with poor outcome in sepsis. However these previous associations were not adjusted for either inflammation or the degree or organ dysfunction [52,53]. We found that in patients presenting with SIRS and sepsis, sVCAM-1 is associated with 28 day mortality, shock and organ dysfunction. However, after adjusting for inflammation using sTNFR-1 concentration, sVCAM-1 was no longer independently associated with 28 day mortality. This result suggests sVCAM-1 is more closely reflective of immune activation compared to other biomarkers of endothelial activation and dysfunction.

While we highlight the independent association of endothelial biomarkers with poor outcomes in sepsis, our study has some limitations. First, this is a single-center study and this may limit the ability to generalize our results to other centers. We did identify expected relationships between inflammatory biomarkers and organ dysfunction and death suggesting that our ICU population is representative of ICU populations at other academic medical centers. Future studies will need to validate our results in an independent, external cohort. Second, there is a degree of missingness that varies for each biomarker. This was due to insufficient sample volumes to measure all biomarkers simultaneously in certain subjects. This cause of missingness is likely to be stochastic and unlikely to be informative and, thus, should bias our results towards the null. Third, we did not include all biomarkers with previous associations with poor outcomes in sepsis such as pro-calcitonin [12,54–57]. This biomarker is not routinely measured for clinical purposes at our institution and will need to be included for comparison to biomarkers of endothelial activation in future studies. Last, we performed multiple tests to identify associations between the various biomarkers and our outcomes. Because the biomarkers are correlated and do not represent truly independent tests, a Bonferroni correction would be too conservative a threshold for determining statistical significance. Nonetheless, the vast majority of associations we report would survive such a correction.

There are studies assessing the associations of endothelial biomarkers, particularly in sepsis, to poor outcome. To our knowledge, we are the first to identify associations in a broader population of patients presenting with SIRS criteria, including both patients with sepsis and patients with other sources of critical illness. Our study also provides evidence supporting an independent role for endothelial activation and dysfunction in SIRS and sepsis pathophysiology. While these robust associations give us further insight into the pathogenesis of SIRS, these biomarkers of disease may have broader implications for clinical use. Future studies will determine whether these biomarkers may be clinically useful for prognostication, triage-decision making, directing therapeutic interventions, and identifying patients early for inclusion in clinical trials of new investigational agents.

## Conclusions

This study shows that biomarkers of endothelial activation and dysfunction are strongly associated with poor outcomes including shock, organ dysfunction, and death in critically ill patients presenting with SIRS. These associations remain robust when adjusted for inflammation suggesting an independent role for endothelial activation and dysfunction in the pathogenesis of SIRS.

## Supporting Information

S1 Table Correlation Between Biomarkers. Table shows spearman rank correlation coefficients between untransformed biomarker concentrations.
(PDF)

S2 Table Subject Characteristics Sepsis versus Sterile Inflammation. Table compares demographics between subjects with sepsis versus sterile inflammation.
(PDF)

S3 Table Comparison of Plasma Biomarkers in Sepsis and Sterile Inflammation (PDF)

S4 Table Multivariate Analysis of Biomarker Association with 28 day mortality in Sepsis. Logistic regression adjusted for age, gender, presence of infection, admitting service (medical vs. surgical), source of admission (outside hospital vs. emergency room), body mass index, smoking status, diabetes mellitus, chronic renal insufficiency, and cirrhosis.
(PDF)

S5 Table Multivariate Analysis of Biomarker Association with 28 day mortality in Sterile Inflammation. Logistic regression adjusted for age, gender, presence of infection, admitting service (medical vs. surgical), source of admission (outside hospital vs. emergency room), body mass index, smoking status, diabetes mellitus, chronic renal insufficiency, and cirrhosis.
(PDF)
