# Supplementary material for: Biomarkers of Endothelial Activation Are Associated with Poor Outcome in Critical Illness
Source: PLoS One. 2015 Oct 22;10(10):e0141251. doi: 10.1371/journal.pone.0141251 (PMC4619633; doi:10.1371/journal.pone.0141251)
Supplement: S1 Table — Table shows spearman rank correlation coefficients between untransformed biomarker concentrations. (PDF) [file pone.0141251.s001.pdf]

S1 Table. Correlation between Biomarkers

	Inflammation:				Endothelial Activation	
	IL-6	IL-8	G-CSF	sTNFR-1	Ang-1	Ang-2
Inflammation:						
IL-8	0.42[a]					
G-CSF	0.59[a]	0.32[a]				
sTNFR-1	0.51[a]	0.49[a]	0.29[a]			
Endothelial Activation:						
Ang-1	-0.18[a]	-0.02	-0.16[a]	-0.27[a]		
Ang-2	0.39[a]	0.24[a]	0.28[a]	0.55[a]	-0.81[a]	
sVCAM-1	0.21[a]	0.35[a]	0.06	0.54[a]	-0.34[a]	0.49[a]

Table shows Spearman's rank correlation coefficients between untransformed biomarker concentrations.

[a] P-value <0.001.
