# Supplementary material for: Biomarkers of Endothelial Activation Are Associated with Poor Outcome in Critical Illness
Source: PLoS One. 2015 Oct 22;10(10):e0141251. doi: 10.1371/journal.pone.0141251 (PMC4619633; doi:10.1371/journal.pone.0141251)
Supplement: S2 Table — Table compares demographics between subjects with sepsis versus sterile inflammation. (PDF) [file pone.0141251.s002.pdf]

S2 Table. Subject Characteristics Sepsis versus Sterile Inflammation

**Characteristics**	**Sepsis (N = 629)**	**Sterile Inflammation (N = 314)**
Patient Age, mean± SD	53.8 ± 15.5	58.6 ± 17.5
Male patients, no. (%)	416 (66.1%)	185 (58.9%)
Caucasian, no. (%)	629 (100%)	314 (100%)
Source of ICU admit, no. (%)		
Medical	372 (59.1%)	144 (45.9%)
Surgical	257 (40.9%)	170 (55.1%)
APACHE III, mean ± SD	54.0 ± 26.7	44.8 ± 24.1
Comorbidities, no. (%)		
Diabetes	169 (26.9%)	64 (20.4%)
Cirrhosis	68 (10.8%)	16 (5.1%)
Chronic Renal Insufficiency	45 (7.2%)	20 (6.4%)
Smoking	357 (56.8%)	181 (57.6%)
BMI, mean ± SD	31.2 ± 11.5	28.9 ± 8.0

SD= Standard Deviation; APACHE III = Acute Physiology and Chronic Health Evaluation III; BMI = Body Mass Index
