# Supplementary material for: Biomarkers of Endothelial Activation Are Associated with Poor Outcome in Critical Illness
Source: PLoS One. 2015 Oct 22;10(10):e0141251. doi: 10.1371/journal.pone.0141251 (PMC4619633; doi:10.1371/journal.pone.0141251)
Supplement: S3 Table — (PDF) [file pone.0141251.s003.pdf]

S3 Table. Comparison of Plasma Biomarkers in Sepsis and Sterile Inflammation

Biomarkers (pg/mL)	Subjects (N)	Sepsis, Median (IQR)	Sterile Inflammation, Median (IQR)	p [a]
Inflammation:				
IL-6	888	134 (63 ,330)	114 (47 ,287)	0.016
IL-8	888	15 (8 ,33)	12 (6 ,24)	8.9×10^{-4}
G-CSF	888	28 (16 ,58)	27 (16 ,51)	0.37
sTNFR-1	888	9304 (5946 ,15531)	7120 (4791 ,13258)	5.1×10^{-6}
Endothelial Activation:				
Ang-1	930	4992 (2184 ,10070)	5624 (2996 ,9228)	0.36
Ang-2	939	16405 (8600 ,33197)	9093 (5526 ,17093)	1.3×10^{-17}
Ang-2/Ang-1	930	4 (1 ,13)	2 (1 ,5)	8.2×10^{-09}
sVCAM-1	939	601 (458 ,869)	494 (398 ,675)	2.7×10^{-10}

IQR= Interquartile Range; IL-6 = Interleukin-6; IL-8 = Interleukin-8; G-CSF = Granulocyte colony stimulating factor; sTNFR-1 = Soluble Tumor Necrosis Factor Receptor-1; Ang-1 = Angiopoietin-1; Ang-2 = Angiopoietin-2; sVCAM-1 = Soluble Vascular Adhesion Molecule-1.

[a] P value for Mann-Whitney U Test
