# Supplementary material for: Biomarkers of Endothelial Activation Are Associated with Poor Outcome in Critical Illness
Source: PLoS One. 2015 Oct 22;10(10):e0141251. doi: 10.1371/journal.pone.0141251 (PMC4619633; doi:10.1371/journal.pone.0141251)
Supplement: S5 Table — Logistic regression adjusted for age, gender, presence of infection, admitting service (medical vs. surgical), source of admission (outside hospital vs. emergency room), body mass index, smoking status, diabetes mellitus, chronic renal insufficiency, and cirrhosis. (PDF) [file pone.0141251.s005.pdf]

S5 Table. Multivariate Analysis of Biomarker Association with 28 Day Mortality in Patients with Sterile Inflammation

	Unadjusted		Adjusted [a]		Apache III adjusted [b]		IL-6 adjusted [c]		sTNFR-1 adjusted [d]	
Biomarkers	OR (95%CI)	p [e]	OR (95% CI)	p	OR (95% CI)	p	OR (95% CI)	p	OR (95% CI)[d]	p
<u>Inflammation:</u>										
IL-6	1.75 (1.40, 2.19)	$1.2x10^{-6}$	2.08 (1.62, 2.66)	$6.4x10^{-9}$	1.75 (1.34, 2.30)	$5.1x10^{-5}$				
IL-8	1.77 (1.45, 2.14)	$9.4x10^{-9}$	1.72 (1.37, 2.15)	$2.5x10^{-6}$	1.76 (1.33, 2.34)	$9.5x10^{-5}$				
G-CSF	1.61 (1.31, 1.97)	$4.6x10^{-6}$	1.86 (1.44, 2.40)	$1.5x10^{-6}$	1.63 (1.26, 2.12)	$2.5x10^{-4}$				
sTNFR-1	2.35 (1.73, 3.19)	$3.9x10^{-8}$	2.53 (1.65, 3.89)	$2.3x10^{-5}$	2.00 (1.20, 3.33)	$7.7x10^{-3}$				
<u>Endothelial Activation:</u>										
Ang-1	0.70 (0.54, 0.90)	$6.3x10^{-3}$	0.75 (0.60, 0.93)	$8.9x10^{-3}$	0.83 (0.64, 1.09)	NS	0.76 (0.59, 0.97)	NS	0.79 (0.60, 1.04)	NS
Ang-2	3.25 (2.14, 4.95)	$3.4x10^{-8}$	3.37 (2.08, 5.45)	$7.6x10^{-7}$	2.68 (1.56, 4.61)	$3.8x10^{-4}$	2.95 (1.70, 5.14)	$1.3x10^{-4}$	3.14 (1.91, 5.17)	$6.8x10^{-6}$
Ang-2/Ang-1	1.73 (1.40, 2.14)	$4.8x10^{-7}$	1.70 (1.36, 2.12)	$2.4x10^{-6}$	1.50 (1.17, 1.92)	$1.4x10^{-3}$	1.58 (1.23, 2.04)	$4.2x10^{-4}$	1.57 (1.24, 1.97)	$1.3x10^{-4}$
sVCAM-1	3.29 (1.97, 5.51)	$5.6x10^{-6}$	3.32 (1.71, 6.46)	$4.1x10^{-4}$	4.11 (1.86, 9.06)	$4.6x10^{-4}$	2.54 (1.23, 5.24)	$2.4x10^{-3}$	1.53 (0.58, 4.05)	NS

OR = Odds Ratio per doubling of biomarker; CI = Confidence Interval; APACHE III = Acute Physiology and Chronic Health Evaluation III; IL-6 = Interleukin-6; IL-8 = Interleukin-8; G-CSF = Granulocyte colony stimulating factor; sTNFR-1 = Soluble Tumor Necrosis Factor Receptor-1; Ang-1 = Angiopoietin-1; Ang-2 = Angiopoietin-2; sVCAM-1 = Soluble Vascular Adhesion Molecule-1.

[a] Logistic regression adjusted for age, gender, presence of infection, admitting service (medical vs. surgical), source of admission (outside hospital vs. emergency room), body mass index, smoking status, diabetes mellitus, chronic renal insufficiency, and cirrhosis

[b] Adjusted for APACHE III and covariates in [a].

[c] Adjusted for Log_2(IL-6) concentration and covariates in [a].

[d] Adjusted for Log_2(sTNFR-1) concentration and covariates in [a].

[e] For the number of tests in this table, a Bonferroni $p<0.05$ is equivalent to $p<1.56x10^{-3}$
